# Case report: Spontaneous coronary artery dissection in a man with Ehlers–Danlos syndrome

**DOI:** 10.3389/fcvm.2022.913259

**Published:** 2022-08-31

**Authors:** Qiao Li, Min Ma, Yong He

**Affiliations:** ^1^Department of Cardiology, West China Hospital, Sichuan University, Chengdu, China; ^2^Department of Cardiology, The Sixth People’s Hospital of Chengdu, Chengdu, China

**Keywords:** spontaneous coronary artery dissection, acute coronary syndrome, Ehlers–Danlos syndrome, imaging, percutaneous coronary revascularization

## Abstract

**Background:**

Spontaneous coronary artery dissection (SCAD), as a medical emergency, represents one of the non-atherosclerotic causes of an acute coronary syndrome (ACS). It often occurs in young and middle-aged females and is a rarity among male patients. Yet, it is easily misdiagnosed or missed even though it has one of the highest in-hospital mortality rates.

**Case summary:**

Here, we present a young male patient admitted to the emergency department of our hospital due to a complaint of acute chest pain. During his hospitalization, we utilized several tools, including imaging modalities, genetic analyses, and clinical strategies, to ensure a proper diagnosis and management of the patient. The results indicated that the patient suffered from SCAD, as well as vascular Ehlers–Danlos syndrome (vEDS). Unfortunately, the patient died of SCAD-related sudden cardiac death (SCD) on the ninth day before the DNA analysis results were obtained. Despite a global effort and huge progress in the clinical characterization of SCAD, as well as patients’ assessments, its pathophysiology remains poorly understood, with a significant recurrence risk and no specific disease-modifying therapy.

**Conclusion:**

Vascular Ehlers–Danlos syndrome, as an inherited connective tissue disorder characterized by congenital connective tissue dysplasia, is a rare and particularly challenging monogenetic disease. It can cause life-threatening changes, including arterial dissections and ruptures, and lead to early death due to COL3A1 pathogenic variants. It is also a rare cause of SCAD. Currently, the gold standard for SCAD diagnosis is coronary angiography (CAG).

## Introduction

Spontaneous coronary artery dissection (SCAD) is an important, rare, and sometimes fatal cause of acute coronary syndrome (ACS) (1), accounting for 1–4% of all the ACS cases. It often occurs in young and middle-aged female patients and is responsible for 25% of ACS in women younger than 50 years (2). Male patients with SCAD are rare and only represent approximately 10% of all the SCAD cases (1). Here, we report a 37-year-old male patient suffering from severe SCAD. The diagnosis was made using a combination of coronary angiography (CAG) and intravascular ultrasound (IVUS). He was also diagnosed with vascular Ehlers–Danlos syndrome (vEDS) type IV according to his clinical features and the identification of a mutation in *COL3A1* through genetic examinations.

## Case presentation

**FIGURE 1:**
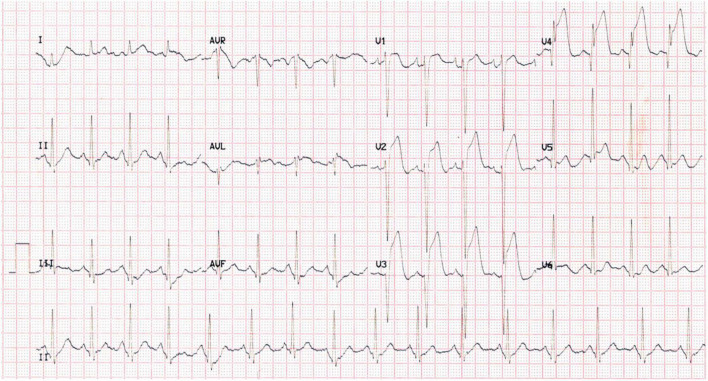
Patient’s ECG on admission.

A 37-year-old male was admitted to our hospital due to a complaint of “a sudden chest pain for 2 h.” The ECG test indicated an acute anterior ST-segment elevation myocardial infarction (STEMI) (Figure 1). Meanwhile, the myocardial injury marker assessments showed elevated troponin T [9,925 ng/l; reference range (RR): 0–14 ng/l], creatine kinase-MB (191.8 ng/ml; RR: < 4.94 ng/ml), and myoglobin (306.6 ng/ml; RR: < 72 ng/ml). We proceeded to perform a transthoracic echocardiography (TTE) test. The results revealed regional wall motion abnormalities within the middle, and lower segments of the ventricular septum and the left ventricular apex, a moderate pericardial effusion, and a 62% left ventricular ejection fraction (LVEF). The differential diagnosis included evolving ACS, acute pulmonary embolism (PE), myocarditis, and pericardial diseases.

The patient had a previous medical history of chronic headaches of unclear origin for more than 20 years. He intermittently took a combination of paracetamol, caffeine, and aspirin in powder form to relieve his symptoms and denied any history of drug abuse. He underwent splenectomy and partial intestinal resection due to a fall-related ruptured spleen and intestine 20 years ago. He also underwent partial liver resection 4 years before the current events due to ruptured liver from another fall injury and was noted to be prone to skin abrasions and subcutaneous ecchymosis.

**FIGURE 2:**
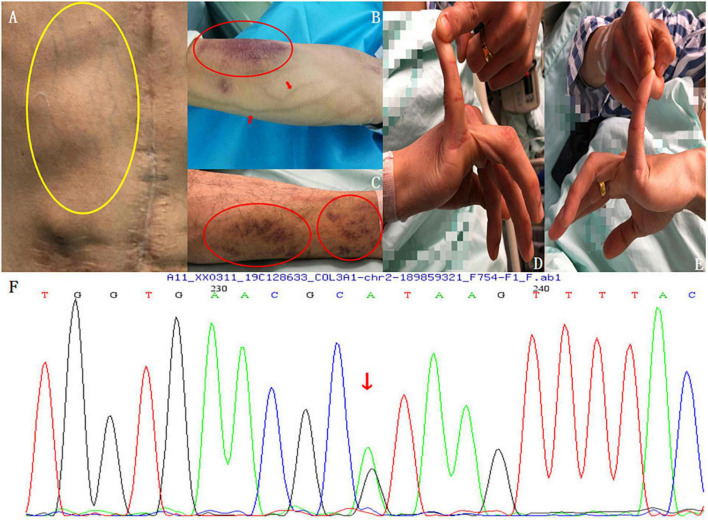
Physical examination findings. **(A)** Thin skin, increased skin elasticity, and obviously exposed subcutaneous veins of the chest wall (yellow circle). **(B)** Visible veins and subcutaneous ecchymosis (red circle) on the forearm. **(C)** Visible subcutaneous ecchymosis on the lower limbs (red circles). **(D,E)** Abnormally increased mobility of the bilateral little finger MCP joints. **(F)** Genetic testing: A heterozygous mutation of c.1347 + 1G > A in the *COL3A1* gene was found and verified using PCR and Sanger sequencing, which led to a protein sequence splicing mutation.

The physical examination showed that the patient had slightly protruding eyes, thin skin, increased skin elasticity, obviously exposed chest and forearm subcutaneous veins, visible subcutaneous ecchymosis on the forearm and lower limbs (Figures 2A–C), and an abnormally increased mobility of the little bilateral finger metacarpophalangeal (MCP) joints (Figures 2D,E). A heterozygous mutation of c.1347 + 1G > A in the *COL3A1* gene was also discovered and verified using PCR and Sanger sequencing, which indicated a protein sequence splicing mutation (Figure 2F). He was then clinically diagnosed with vEDS based on the synthesis of imaging techniques, medical history, clinical manifestations, and DNA sequence analyses.

**FIGURE 3:**
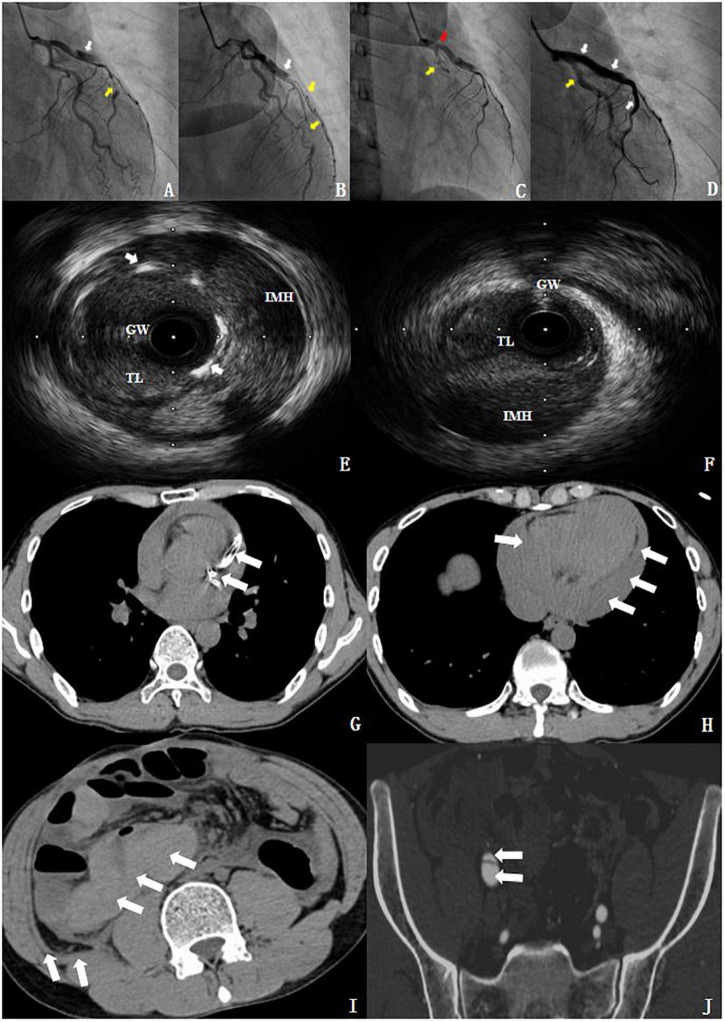
Imaging findings. **(A,B)** Coronary angiography (CAG). The white arrow shows a type 1 dissection at the proximal segments of LAD. The yellow arrow indicated a type 2 dissection at the middle segments of the LAD and the diagonal branch. **(C)** The patient showed progression of coronary artery dissection during the CAG. The red arrow demonstrates that the dissection involved the LM and the yellow arrow indicates a type 4 dissection of the LCX. **(D)** After revascularization. The white arrow shows the TIMI flow grade 3 at the LM and LAD. The yellow arrow indicates the TIMI flow grade 3 at the LCX. **(E)** IVUS showed a massive IMH from the media external to the LAD stent; the white arrow indicated the stent struts. **(F)** The true lumen (TL) of the median LCX segment was severely deformed by the dissection and the massive media layer hematoma. GW, guidewire. **(G,H)** The chest CT revealed a stent in the left anterior descending coronary artery and hemopericardium. **(I,J)** The abdominal and pelvic CT showed intestinal wall swelling, hematocele, retroperitoneal hematocele, and an iliac vascular dissection (see section “Supplementary material”).

The mentioned findings prompted an emergency selective CAG *via* the right radial artery, which indicated a dissection located between the proximal and middle segments of the left anterior descending (LAD) branch (Figures 3A,B). The results indicated a progression of the coronary artery dissection involving the left main (LM) trunk and the left circumflex branch (LCX), which caused an LCX occlusion of the proximal segment (type 4 dissection of the LCX) (Figure 3C) with the Thrombolysis in Myocardial Infarction (TIMI) flow grade 0 during CAG. Accordingly, the patient developed hemodynamic instability and immediately underwent percutaneous coronary intervention (PCI). Intravascular ultrasound (IVUS) further confirmed the coronary artery dissection and guided the revascularization process. It showed a massive intramural hematoma (IMH) from the media external to the LAD stent, with the true vascular lumen of the median LCX segment being severely deformed by the dissection and the massive media layer hematoma (Figures 3E,F). During the procedure, three drug-eluting stents (DESs) were placed from the LAD to the LM (the stents were Promus PREMIER™ 2.5 mm × 38 mm, 3.5 mm × 32 mm, and 4.0 mm × 24 mm, from Boston Scientific Corporation, United States, respectively). These stents were overlapped after revascularization with another stent at the LCX (the stent was Promus PREMIER™ 2.75 mm × 20 mm, Boston Scientific Corporation, United States) and inflated (12–16 atm inflation pressure) for deployment. Finally, the results indicated that hemodynamic stability was successfully achieved, and the LCX improved to the TIMI flow grade 3 (Figure 3D). The patient was transferred to the cardiac care unit for treatment following the procedure. Dual antiplatelet therapy with 100 mg of aspirin and 75 mg of clopidogrel was administered once daily since the subject underwent a DES implantation.

To further clarify and eliminate potential non-coronary artery disease-related problems, we proceeded to perform a postoperative chest, abdominal, and pelvic enhanced CT examination. The chest CT revealed a stent in the left anterior descending coronary artery and hemopericardium (Figures 3G,H). Meanwhile, the abdominal and pelvic CT showed intestinal wall swelling, hematocele, retroperitoneal hematocele, and an iliac vascular dissection (Figures 3I,J). Unfortunately, the patient suffered from a sudden cardiac arrest, and all the resuscitation attempts failed. He, unfortunately, died of sudden cardiac death (SCD) on the ninth day of the index SCAD event, before the DNA analysis results were obtained. Additionally, the bedside echocardiography showed a large amount of pericardial effusion. According to the description of the doctor on duty at that time, the patient suffered from chest tightness and hypovolemic shock, and the patient died before pericardiocentesis. We speculated that possible causes of death included left ventricular rupture or coronary artery perforation after myocardial infarction (MI), as well as SCAD, accompanied by rupture and bleeding. Because in China, an autopsy is not accepted by the majority of people, because people hope that they will be able to have a complete body after death. The same as the cases reported in our article, in which the family refused autopsy. Interestingly, there have been few reports of deaths imputed to a fatal vEDS complication (3, 4). Further probing showed that the patient had an unremarkable family history.

## Discussion

SCAD often forms a continuously growing false lumen outside of the media layer of the coronary artery wall. Yet, the mechanism of SCAD occurrence is unclear. CAG is the gold standard for the diagnosis of SCAD, and optical coherence tomography (OCT), as well as intravascular ultrasound (IVUS) as essential tools for its confirmation (5) even though extra caution, is needed when imaging patients with such a condition. The cause of SCAD is unknown, but probably includes factors related to gender, especially in perinatal women (6) (the mechanism of effect is unknown), as well as fibromuscular dysplasia, systemic inflammatory condition, and hormone therapies.

Ehlers–Danlos syndrome (EDS) is an inherited connective tissue disorder characterized by congenital connective tissue dysplasia. Primarily occurring in young and middle-aged women, SCAD is an important, rare, and fatal cause of ACS, with none to few conventional cardiovascular risk factors (7). Contrarily, male patients with SCAD are extremely rare (7, 8), and notably, they more often cite a physical stressor as the cause of the disease instead of anxiety or depression. As one of the predisposing conditions for SCAD, vEDS is a rare and particularly challenging monogenetic disease caused by a mutation in the *COL3A1* gene coding for type III procollagen. Patients with vEDS are at risk of vascular, intestinal, and uterine ruptures (9). SCAD is not common among males, and vEDS is rarer among this group.

The prevalence of vEDS is approximately 1 out of 25,000 people (10), and its primary diagnostic criteria include easy bruising, transparent skin, characteristic facial features, as well as arterial, intestinal, or uterine rupture. The mechanism of SCAD initiation remains unclear since only 1–2% of SCAD has been related to inherited connective tissue disorders (7) (Table 1). But, there are currently two potential theories. The first theory involves the “outside-in” mechanism, stipulating that microvascular ruptures lead to the formation of intramural hematoma on the coronary artery wall. Meanwhile, the second theory supports the “inside-out” mechanism, stating that a disrupted continuity or rupture of the endothelium and intima leads to blood penetration through the internal elastic lamina and accumulation in the media (11) (Tables 1, 2). Non-invasive imaging, such as CT coronary angiography (CTCA) and MR angiography, is preferred since invasive angiographies in patients with SCAD have been associated with an increased risk of iatrogenic dissections (1).

**TABLE 1:** Spontaneous coronary artery dissection (SCAD).

Etiology of non-atherosclerotic SCAD	
**Predisposing arteriopathy**	
A. Fibromuscular dysplasia	
B. Connective tissue disorder: Marfan’s syndrome, Ehlers–Danlos syndrome, Cystic medial necrosis	
C. Systemic inflammation: Systemic lupus erythematosus, Crohn’s disease, Sarcoidosis	
D. Pregnancy	
E. Coronary artery spasm	
F. Idiopathic	
G. Hormonal therapy	
**Precipitating stress events**	
A. Intense emotional stress	
B. Intense exercise	
C. Intense Valsalva-type activities	
D. Labor and delivery	
E. Cocaine, amphetamines, met-amphetamines, β-HCG	

**Angiographic classification for SCAD**	

**Type**	**Characteristics**
Type 1	Dissection with visible linear “flap” or dual lumen often associated with contrast hold-up
Type 2	Type 2a dissection with no visible “flap” and distal reconstitution of normal vessel architecture Type 2b dissection with no visible “flap” and no distal reconstitution
Type 3	Focal or tubular stenosis (length typically < 20 mm)that mimics atherosclerosis
Type 4	With a total, usually of a distal vessel, vessel occlusion and no cardiac embolic source and there is subsequent evidence of complete vessel healing in keeping with the natural history of SCAD

**TABLE 2:** Intracoronary imaging of spontaneous coronary artery dissection (SCAD) by IVUS/OCT/CT/CMR and management.

Multimodality imaging	Axial resolution (um)	Advantage	Disadvantage or limitation
Intravascular ultrasound (IVUS)	150	A. Blood clearance is not required B. IVUS has greater depth penetration	Poor spatial resolution
Optical coherence tomography (OCT)	10–20(15)	A. Higher spatial resolution B. Provides a finer depiction of vessel’s wall characteristic images of SCAD	A. Necessitates blood clearance requiring a high pressure contrast injection B. Depth penetration
Computed tomography coronary angiography (CTCA)		Non-invasive	A. Lower spatial resolution and temporal resolution B. Affected by breathing and heart rate
Cardiac magnetic resonance (CMR)		Non-invasive, safe and non-radiating	A. High price is not easy to popularize B. Affected by breathing and heart rate

**Management**			

A. Conservative management			
B. Percutaneous coronary intervention (PCI)			
C. Coronary artery bypass grafting (CABG)			
D. Adjunctive supportive devices and transplant			
E. Medical management (Decide whether to use according to individual’s situation) a. Thrombolysis: Thrombolysis is contraindicated for the acute management of SCAD b. Antiplatelet therapies: The use of antiplatelet therapies and the duration of treatment remains an area of controversy and divergent practice in SCAD c. Anticoagulant therapies: Limited to acute phase during revascularization procedures while chronic use should be restricted to situations where there is an unequivocal clinical indication (such as left ventricular thrombus or thromboembolism) d. Angiotensin converting enzyme inhibitors/angiotensin receptor antagonists: SCAD patients with significant impairment of left ventricular systolic function should follow current guidelines e. Mineralocorticoid receptor antagonists: SCAD patients with significant impairment of left ventricular systolic function should follow current guidelines f. Beta-blockers: SCAD patients with significant impairment of left ventricular systolic function should follow current guidelines g. Vasodilator therapies: Reserved for the empirical treatment of chest pain during the acute phase and recurrent chest pain following the index event h. Statins: Reserved for patients with conventional indications for treatment independent of their SCAD event i. Contraception and hormone replacement therapy: A reasonable strategy may be to avoid hormonal contraception where possible. In patients with recurrent cyclical chest pain following SCAD, low dose local hormone delivery intrauterine contraceptive devices have been anecdotally reported to be helpful			
F. Cardiac rehabilitation			

As one of the predisposing conditions for SCAD, EDS is an inherited connective tissue disorder, manifesting as congenital connective tissue dysplasia. EDS is classified into six subtypes. Type IV, as the vascular type, also known as vEDS, is an extremely rare autosomal dominant genetic disorder characterized by changes in the vascular structure due to a mutation in the *COL3A1* gene coding for type III procollagen. It leads to increased brittleness of the connective tissue and fatal complications (for example, vascular ruptures, organ ruptures, and fistulae formation) (12). Sadly, vEDS prognosis is the worst among all the types of EDS due to previously mentioned complications (13). SCAD is not common among males, and it is even extremely rare for them to be diagnosed with vEDS. Additionally, a clinical diagnosis of vEDS is often difficult without genetic testing for *COL3A1* mutations.

SCAD treatment during the acute is determined according to the patient’s clinical conditions and the TIMI flow grade as shown by CAG. According to present guidelines, conservative therapy is the first-line recommended treatment for patients with SCAD. Additionally, a cutting balloon (CB) angioplasty might be an alternative option for SCAD revascularization since it permits the fenestration of the false lumen to allow communication and back-bleed of the intramural hematoma into the true lumen (14). Based on our experience, the distal position of the vascular dissection should be determined under the guidance of IVUS. In this case, the distal and proximal vessels were cut. Experience has shown that it can be cut at multiple positions, and the relatively safe ratio of cutting balloon to vessel diameter is 0.7:1.

Of course, SCAD patients’ coronary revascularization is extremely challenging due to coronary arterial fragility in these patients and the tendency for dissections. That is why, conservative medical management strategies are preferred in clinically stable patients with a maintained coronary flow. No prospective randomized data are available to guide medical management and whether the standard ACS pharmacological treatment is beneficial in SCAD is unclear. Thus, these patients, based on national guidelines, are often treated empirically with medicines, such as β-blockers, angiotensin-converting enzyme inhibitors or angiotensin receptor blockers, and antiplatelet drugs (7). Of the mentioned drugs, celiprolol is a third-generation cardioselective β1 blocker with a β2 agonist vasodilatory effect. Its preventive effects on arterial mortality and morbidity have been evidenced in one clinical trial [the Beta-Blockers in Ehlers–Danlos Syndrome Treatment (BBEST)]. However, the study had certain limitations. Furthermore, a theoretical risk of vasospasm exacerbation exists with β-blocker treatments (15).

The decision to proceed with PCI or coronary artery bypass surgery (CABG) depends on the patient’s acute presentation and technical considerations, which widely differ among clinicians. PCI with stents is recommended in cases where a major coronary artery is involved or if the patient is unstable. Meticulous angiographic techniques should be practiced in patients with SCAD. However, deep catheter intubations should be avoided, with special care given to pressure dampening and coronary injections. CABG in SCAD is generally only used as a bailout strategy and is reserved for failed PCI with ongoing ischemia or infarction of a significant at-risk myocardial territory (for example, LM dissections with ongoing ischemia/infarction) (7).

## Conclusion

In summary, clinicians need to be aware of vEDS as a potential SCAD cause and its various presentations. Additionally, cardiovascular genetic testing and targeted management should be considered for patients with SCAD, especially in younger subjects.

## Data availability statement

The datasets for this article are not publicly available due to concerns regarding participant/patient anonymity. Requests to access the datasets should be directed to the corresponding author.

## Ethics statement

The studies involving human participants were reviewed and approved by the Biomedical Research Ethics Committee, West China Hospital. The patients/participants provided their written informed consent to participate in this case study. Written informed consent was obtained from the individual(s) for the publication of any potentially identifiable images or data included in this article.

## Author contributions

QL and MM participated in the design, collected the data, and drafted the manuscript. YH helped to revise the manuscript critically for important intellectual content. All authors have read and approved the final version of the manuscript.
